# Supplementary material for: Role of JAK-STAT Pathway in Broiler Chicks Fed with Chestnut Tannins
Source: Animals (Basel). 2021 Jan 29;11(2):337. doi: 10.3390/ani11020337 (PMC7911350; doi:10.3390/ani11020337)

SUPPLEMENTARY MATERIALS:

Figure S1. Calculated composition of corn/soy starter diets. The total basal diet contained 1,365 kcal/lb.

**Ingredients**	**%**
Corn	59.81
SBM 48%	33.84
Monocalcium phosphate 21	1.56
Soy oil	2.09
Choline chloride	0.10
Limestone	1.56
Salt	0.33
L-lysine HCL	0.19
DL-methionine	0.28
Vitamin premix	0.13
Mineral premix	0.05
L-Threonine	0.05
**Calculated nutrients, %**	99.99
Protein	22.00
Calcium	0.90
Available phosphorus	0.45
AMEn (Kcal/lb)	1,365.00
Digestible methionine	0.59
Digestible total sulfa amino acid	0.88
Digestible threonine	0.77
Digestible lysine	1.18
Choline	1,256.87
Sodium	0.16
Potassium	0.84
Chloride	0.20

Figure S2A-C. STRING-db network of interactions. Purple circles indicate the JAK-STAT peptides affiliated to the pathway; the other colors indicate regulatory pathways affecting the JAK-STAT pathway: (A) Protein-protein interactions between proteins included in the day 4 chicken immune peptide array and metabolic peptide array; (B) Protein-protein interactions between proteins included in the day 6 chicken immune peptide array and metabolic peptide array; (C) Protein-protein interactions between proteins included in the day 10 chicken immune peptide array and metabolic peptide array

A

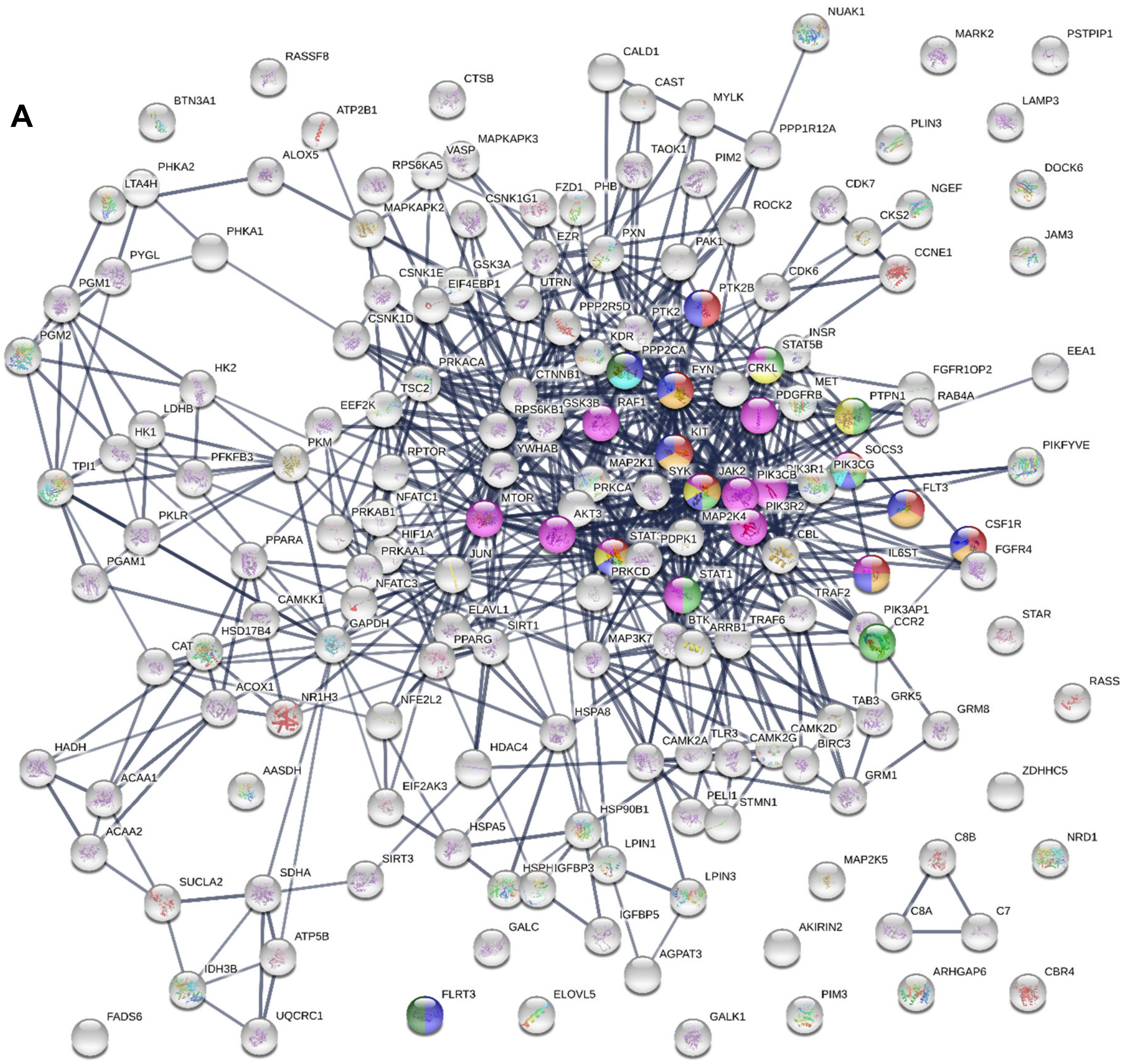
NUAK1
MARK2
PSTPIP1
RASSF8
CALD1
CTSB
CAST
MYLK
BTN3A1
ATP2B1
LAMP3
PLIN3
PPP1R12A
MAPKAPK3
ALOX5
PHKA2
VASP
RPS6KA5
TAOK1
PIM2
DOCK6
LTA4H
CSNK1G1
FZD1
PHB
CDK7
NGEF
MAPKAPK2
ROCK2
PHKA1
EZR
PXN
CKS2
JAM3
PYGL
GSK3A
PAK1
CCNE1
CSNK1E
PGM1
EIF4EBP1
UTRN
CDK6
PTK2B
CSNK1D
PPP2R5D
PTK2
INSR
PGM2
KDR
STAT5B
EEA1
PPP2CA
HK2
PRKACA
CTNNB1
FYN
CRKL
MET
FGFR1OP2
TSC2
EEF2K
GSK3B
RAF1
PDGFRB
RAB4A
LDHB
RPS6KB1
PTPN1
HK1
KIT
SOCS3
PIKFYVE
PKM
YWHAB
RPTOR
MAP2K1
SYK
JAK2
PIK3CB
PIK3R1
PIK3CG
TPI1
PFKFB3
PRKCA
FLT3
NFATC1
MTOR
AKT3
MAP2K4
PIK3R2
CSF1R
PKLR
PRKAB1
HIF1A
PDPK1
CBL
IL6ST
FGFR4
PPARA
JUN
STAT
PRKAA1
PRKCD
STAT1
PGAM1
NFATC3
TRAF2
PIK3AP1
CAMKK1
ELAVL1
STAR
HSD17B4
GAPDH
SIRT1
BTK
ARRB1
TRAF6
CCR2
CAT
PPARG
MAP3K7
RASS
ACOX1
NR1H3
NFE2L2
HSPA8
TAB3
GRK5
GRM8
HADH
HDAC4
CAMK2A
TLR3
CAMK2G
CAMK2D
BIRC3
AASDH
EIF2AK3
GRM1
ZDHHC5
ACAA1
PELI1
STMN1
HSP90B1
ACAA2
HSPA5
LPIN1
C8B
NRD1
SIRT3
MAP2K5
SUCLA2
SDHA
HSPH
IGFBP3
LPIN3
C8A
C7
ATP5B
GALC
IGFBP5
AKIRIN2
IDH3B
AGPAT3
ARHGAP6
CBR4
FLRT3
ELOVL5
PIM3
FADS6
UQCRC1
GALK1

B

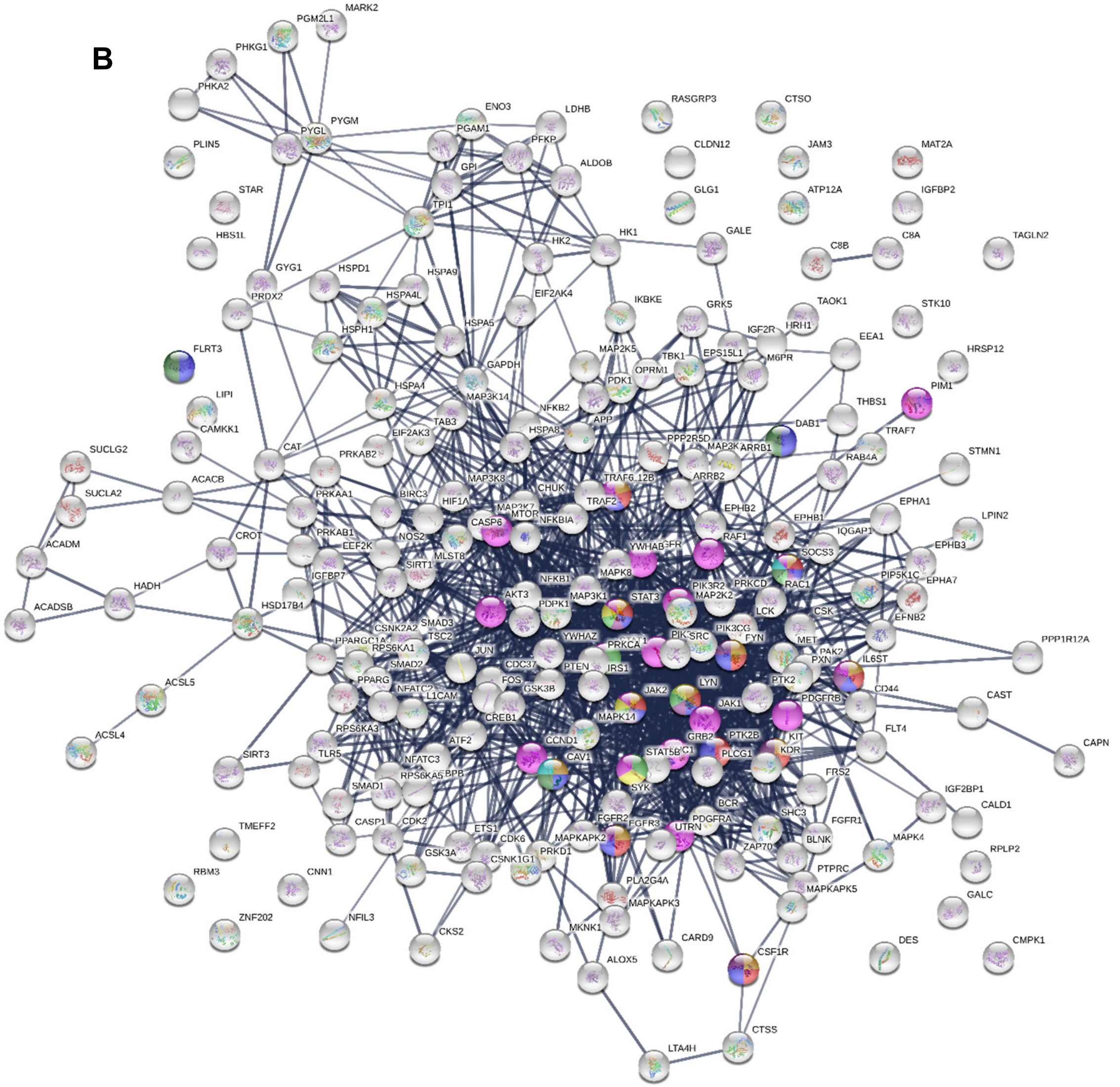

MARK2
PGM2L1
PHKG1
PHKA2
PYGL
PYGM
PLIN5
STAR
HBS1L
GYG1
PRDX2
FLRT3
LIPI
CAMKK1
SUCLG2
SUCLA2
ACACB
ACADM
ACADSB
HADH
CROT
HSD17B4
ACSL5
ACSL4
SIRT3
TMEFF2
RBM3
ZNF202
CNN1
NFIL3
CKS2
ENO3
PGAM1
PFKP
LDHB
ALDOB
GPI
TPI1
HK1
HK2
HSPD1
HSPA9
HSPA4L
HSPH1
HSPA5
EIF2AK4
GAPDH
HSPA4
MAP3K14
EIF2AK3
TAB3
HSPA8
NFKB2
CAT
PRKAB2
PRKAA1
PRKAB1
EEF2K
IGFBP7
BIRC3
HIF1A
NOS2
SIRT1
MLST8
CASP6
MTOR
NFKBIA
CHUK
TRAF2
MAP3K8
RASGRP3
CTSO
CLDN12
JAM3
MAT2A
GLG1
ATP12A
IGFBP2
GALE
C8B
C8A
TAGLN2
IKBKE
GRK5
IGF2R
HRH1
TAOK1
STK10
EEA1
HRSP12
MAP2K5
TBK1
EPS15L1
M6PR
PDK1
OPRM1
APP
PIM1
THBS1
DAB1
TRAF7
PPP2R5D
ARRB1
RAB4A
STMN1
ARRB2
EPHB2
EPHA1
LPIN2
EPHB1
IQGAP1
EPHB3
SOCS3
PIP5K1C
EPHA7
EFNB2
RAC1
PRKCD
RAF1
YWHAB
MAPK8
NFKB1
MAP3K1
STAT3
PIK3R2
MAP2K2
AKT3
PDPK1
CSNK2A2
SMAD3
PPARGC1A
RPS6KA1
TSC2
JUN
YWHAZ
PRKCA
PIK3CG
SRC
FYN
LCK
CSK
MET
PAK2
PXN
IL6ST
PPP1R12A
SMAD2
PPARG
NFATC2
L1CAM
CDC37
FOS
GSK3B
PTEN
IRS1
JAK2
LYN
PTK2
JAK1
PDGFRB
CD44
CAST
CAPN
RPS6KA3
CREB1
ATF2
MAPK14
CCND1
GRB2
PTK2B
KIT
FLT4
TLR5
NFATC3
RPS6KA5
CAV1
STAT5B
PLCG1
KDR
SMAD1
SYK
FRS2
IGF2BP1
CALD1
CASP1
CDK2
BCR
SHC3
FGFR1
FGFR2
FGFR3
PDGFRA
UTRN
ETS1
CDK6
MAPKAPK2
ZAP70
BLNK
MAPK4
RPLP2
GSK3A
CSNK1G1
PRKD1
PTPRC
MAPKAPK5
PLA2G4A
MAPKAPK3
MKNK1
GALC
CARD9
DES
CMPK1
CSF1R
ALOX5
CTSS
LTA4H

C

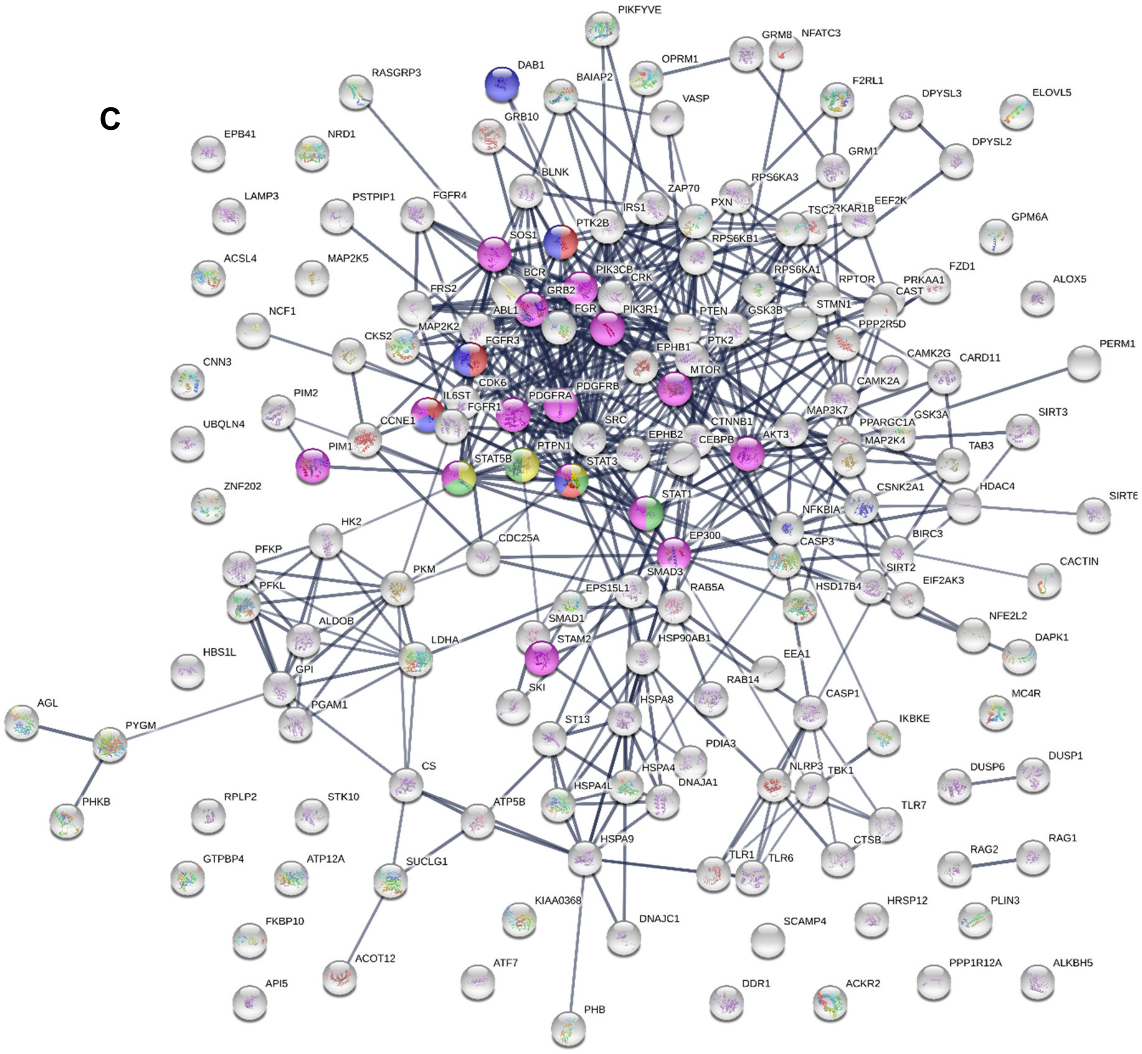
PIKFYVE
GRM8
NFATC3
DAB1
OPRM1
RASGRP3
BAIAP2
F2RL1
DPYSL3
ELOVL5
VASP
GRB10
EPB41
NRD1
DPYSL2
GRM1
BLNK
LAMP3
PSTPIP1
FGFR4
ZAP70
RPS6KA3
EEF2K
IRS1
PXN
TSC2
KAR1B
GPM6A
PTK2B
SOS1
RPS6KB1
ACSL4
MAP2K5
BCR
PIK3CB
CRK
RPS6KA1
RPTOR
FZD1
PRKAA1
ALOX5
CAST
FRS2
GRB2
NCF1
ABL1
FGR
PIK3R1
PTEN
GSK3B
STMN1
PPP2R5D
MAP2K2
CKS2
FGFR3
EPHB1
PTK2
CAMK2G
CARD11
PERM1
CNN3
CDK6
MTOR
CAMK2A
PIM2
IL6ST
PDGFRA
PDGFRB
FGFR1
CCNE1
SRC
CTNNB1
MAP3K7
GSK3A
PPARGC1A
SIRT3
UBQLN4
PIM1
PTPN1
EPHB2
CEBPB
AKT3
MAP2K4
TAB3
STAT5B
STAT3
ZNF202
STAT1
CSNK2A1
HDAC4
SIRT
NFKBIA
HK2
PFKP
CDC25A
EP300
CASP3
BIRC3
SIRT2
CACTIN
PKM
SMAD3
EIF2AK3
PFKL
EPS15L1
RAB5A
HSD17B4
ALDOB
SMAD1
NFE2L2
DAPK1
LDHA
STAM2
HSP90AB1
HBS1L
EEA1
GPI
SKI
RAB14
CASP1
MC4R
PGAM1
AGL
PYGM
ST13
HSPA8
IKBKE
PDIA3
DUSP6
DUSP1
CS
HSPA4
NLRP3
TBK1
DNAJA1
RPLP2
STK10
ATP5B
HSPA4L
TLR7
PHKB
RAG2
RAG1
HSPA9
CTSB
TLR1
TLR6
GTPBP4
ATP12A
SUCLG1
KIAA0368
HRSP12
PLIN3
FKBP10
SCAMP4
DNAJC1
ACOT12
ATF7
PPP1R12A
ALKBH5
API5
DDR1
ACKR2
PHB

Figure S3A-C. mRNA expression of cecal mRNA from experimental chicks fed 1% ChT inclusion diet, determined by quantitative RT-PCR. (A) Day 4 fold changes by cytokines tested comparing averaged 1% ChT inclusion group with control group; (B) Day 6 fold changes by cytokines tested comparing averaged 1% ChT inclusion group with control group; (C) Day 10 fold changes by cytokines tested comparing averaged 1% ChT inclusion group with control group. Data represent the median values from two separate experiments. * = significantly different from the controls (p ≤0.05).

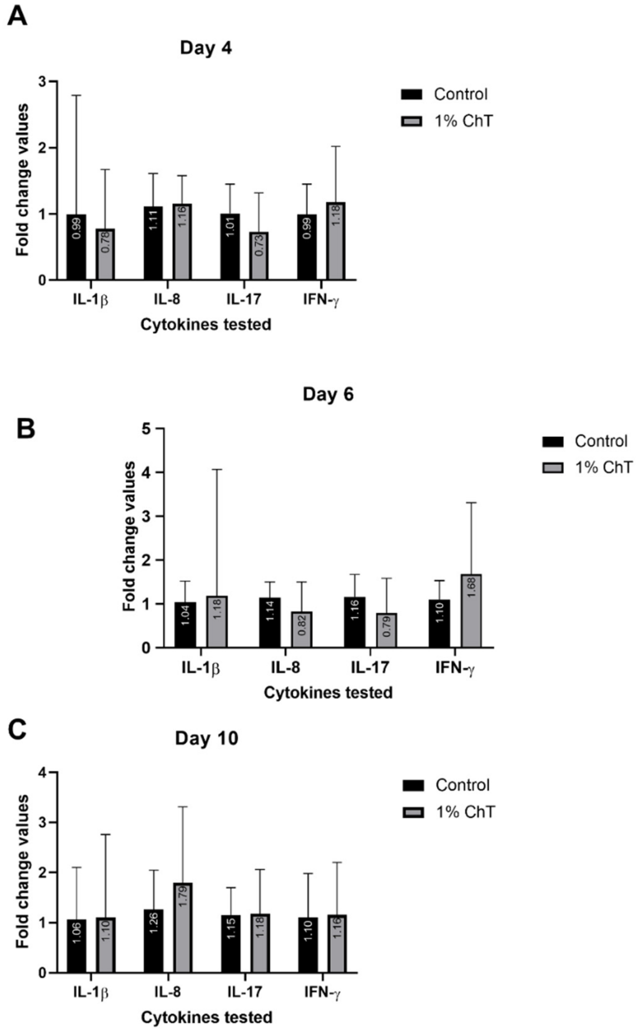

Figure S4. The phosphorylation of STAT proteins activates the initiation of gene transcription. The black arrows indicate phosphorylation, and the red arrow indicates inhibitory action.

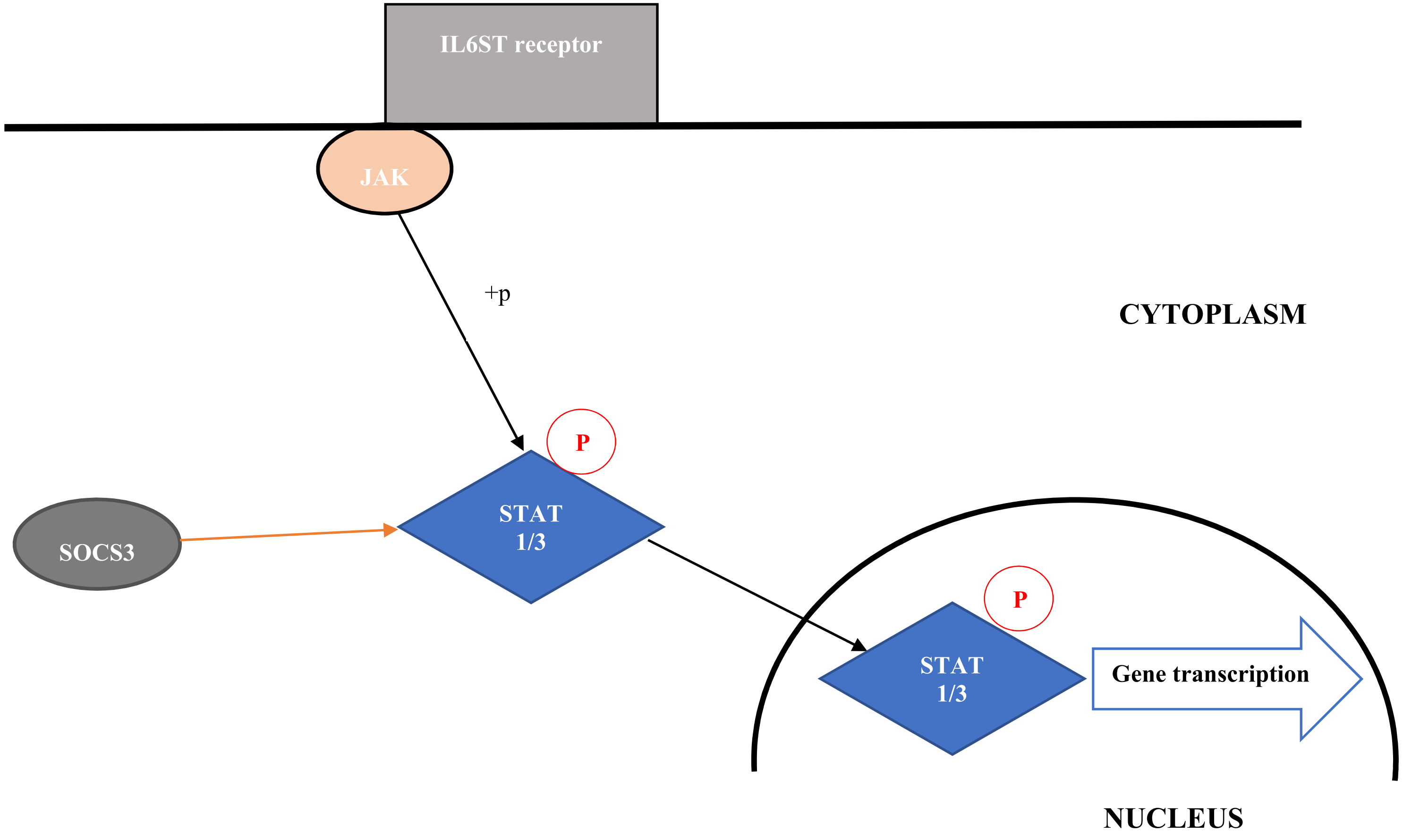

Supplement: Supplementary file 1 [file animals-11-00337-s001.pdf]
